# RANKL and OPG and their influence on breast volume changes during pregnancy in healthy women

**DOI:** 10.1038/s41598-020-62070-3

**Published:** 2020-03-20

**Authors:** Marius Wunderle, Matthias Ruebner, Lothar Häberle, Eva Schwenke, Carolin C. Hack, Christian M. Bayer, Martin C. Koch, Judith Schwitulla, Ruediger Schulz-Wendtland, Ivona Kozieradzki, Michael P. Lux, Matthias W. Beckmann, Sebastian M. Jud, Josef M. Penninger, Michael O. Schneider, Peter A. Fasching

**Affiliations:** 1 Department of Gynecology and Obstetrics, Comprehensive Cancer Center Erlangen-EMN, Erlangen University Hospital, Friedrich Alexander University of Erlangen–Nuremberg, Erlangen, Germany; 2 Biostatistics Unit, Department of Gynecology and Obstetrics, Comprehensive Cancer Center Erlangen-EMN, Erlangen University Hospital, Friedrich Alexander University of Erlangen–Nuremberg, Erlangen, Germany; 3 Institute of Diagnostic Radiology, Comprehensive Cancer Center Erlangen-EMN, Erlangen University Hospital, Friedrich Alexander University of Erlangen–Nuremberg, Erlangen, Germany; 4 Department of Medical Genetics, Life Sciences Institute, University of British Columbia, Vancouver, Canada; 5 Institute of Molecular Biotechnology of the Austrian Academy of Sciences (IMBA), Vienna, Austria; 6 Klinik für Gynäkologie und Geburtshilfe, Frauenklinik St. Louise, Paderborn, St. Josefs-Krankenhaus, Salzkotten, Germany

**Keywords:** Cancer prevention, Predictive markers, Translational research, Breast cancer

## Abstract

Breast cancer risk is reduced by number of pregnancies and breastfeeding duration, however studies of breast changes during or after pregnancy are rare. Breast volume changes – although not linked to breast cancer risk – might be an interesting phenotype in this context for correlative studies, as changes of breast volume vary between pregnant women. Serum receptor activator of nuclear factor kappa B ligand (RANKL) and its antagonist osteoprotegerin (OPG) were measured prospectively before gestational week 12, and three-dimensional breast volume assessments were performed. A linear regression model including breast volume at the start of pregnancy, RANKL, OPG, and other factors was used to predict breast volume at term. The mean breast volume was 413 mL at gestational week 12, increasing by a mean of 99 mL up to gestational week 40. In addition to body mass index and breast volume at the beginning of pregnancy, RANKL and OPG appeared to influence breast volume with a mean increase by 32 mL (*P* = 0.04) and a mean reduction by 27 mL (*P* = 0.04), respectively. Linking the RANKL/RANK/OPG pathway with breast volume changes supports further studies aiming at analysing breast changes during pregnancy with regard to breast cancer risk.

## Introduction

Many risk factors for breast cancer are either characteristics of a woman’s reproductive history or are indirectly related to it^1,2^. The number of pregnancies and the duration of breastfeeding appear to play a pivotal role in this context^3^. Data from a large case–control study provide evidence that in Western industrialized countries, the cumulative lifetime risk of 6.3% up to the age of 70 could be reduced to 2.7% if the average number of full-term pregnancies was 6.5 instead of 2.5 and if the duration of breastfeeding was 24 months per lifetime instead of 8.7 months^3^.

Interestingly, pregnancies appear to have an influence on breast cancer risk that is dependent on a woman’s age at the first full-term pregnancy and on aging. In postmenopausal women, the protective effect of previous pregnancies is well established, regardless of age at the first full-term pregnancy. However, women who have their first child after the age of 35 have a transient increase in the risk of breast cancer up to 15 years after the pregnancy, in comparison with women of similar age without a pregnancy^4^. Although the effects of pregnancy and breastfeeding on breast cancer risk have been described in many epidemiological studies, little is known about macroscopic, microscopic or molecular changes during pregnancy that mediate the risk modification caused by pregnancies.

Mammographic density as a risk factor for breast cancer correlates inversely with the number of pregnancies, as has been shown in several cross-sectional and case–control studies^5–11^. In a retrospective analysis with mammograms available before and shortly after a pregnancy, our group has shown that mammographic density decreases on average after pregnancy, although there is a very variable response to pregnancy in each woman, with some women losing up to almost 50% of mammographic density after pregnancy, while others even have a 15% increase^12^.

In animal models, mammary gland changes during pregnancy have been reported to be progesterone-mediated, through receptor activator of nuclear factor kappa B (RANK), RANK ligand (RANKL), and its antagonist osteoprotegerin (OPG)^13,14^. This pathway has also been implicated in the development of progestin-driven breast cancer, and inhibiting it can prevent carcinogenesis in the breast^15,16^. Mammographic density has been associated with progesterone receptor–positive breast cancer^17^, and high calcium levels have been linked with lower mammographic density^18^ — supporting the hypothesis that RANKL/RANK/OPG signalling may be important for breast changes and potential alterations in the risk of breast cancer.

Measuring breast changes during pregnancy in healthy women is challenging, since imaging methods that involve radiation exposure, such as mammography, cannot be performed without good reason (i.e. for the purpose of studying density changes) and other techniques such as magnetic resonance imaging are too time-consuming and cost-intensive. Our group therefore previously established a method of measuring breast volumes using a three-dimensional (3D) assessment technique^19^. A pilot study showed that women have a mean increase of 95 mL in breast volume during a full-term pregnancy^20^, but also that there is a relevant variability in breast volume changes between pregnant women.

Breast volume changes during pregnancy have not been studied yet with regard to their association with later breast cancer risk. Nevertheless, it might be helpful to connect this easy-to-measure phenotype with molecular pathways that are linked to breast cancer pathogenesis. Therefore, aim of the present study was to assess the influence of RANKL and OPG, as measured in the serum of healthy pregnant women at gestational week 12, on changes in breast volume during pregnancy.

## Patients and methods

### Patients

The Clinical Gravidity Association Trial and Evaluation (CGATE) programme is a prospective observational trial in pregnant women that has several study aims. In addition to pregnancy-related outcomes, the research programme also aims to investigate the physical changes that women experience during pregnancy. Changes in the breast are among these. From November 2009 to December 2012, a total of 298 women were included in the CGATE programme. They had to be at least 18 years old and diagnosed with an intact pregnancy no later than gestational week 13. They were followed up prospectively, with an optional visit at week 23 and a mandatory visit at the end of the pregnancy. For the present study, women were excluded in the following hierarchical order: 26 women had to be excluded because breast imaging was not carried out at either time point; six women had to be excluded because a second assessment was not available after the first had been done; 160 women had to be excluded because they had not yet reached the study visit within two weeks before calculated pregnancy due date; and six women had to be excluded because no serum or insufficient serum was available (e.g., due to hemolysis) until gestational week 13 at study entry. A total of 100 women ultimately participated in the study. Conduct of the CGATE study as described here has been previously published by our group^20^, without available data on serum RANKL and OPG at this time.

### Documentation

The pregnant women were asked to complete a structured questionnaire to provide common epidemiological information on entry into the study. They were also provided with a pregnancy diary that had to be completed every month up to the end of the pregnancy. The questions were concerned with lifestyle, body weight, nutrition, and pregnancy-related diseases, as well as restless leg syndrome and depression during pregnancy. The maternity record (*Mutterpass*), which is mandatory in Germany and documents the obstetrician’s monitoring of the pregnancy, and the patient chart at birth were also used to document the characteristics of the woman, child, and pregnancy. Documentation was performed as part of our former pilot study^20^.

### Assessment of breast volumes

Breast volumes were assessed at study entry (around gestational week 12–13, subsequently referred to as week 12) and at the end of the pregnancy (around gestational week 38–40, subsequently referred to as week 40). In brief, a 3D image was taken during inspiration with the patient in an upright position, using an optical 3D sensor (BreastSCAN3D; 3D-Shape Ltd., Erlangen, Germany) for acquisition of the breast surfaces. After acquisition of the breast surface data, a calibrated digital texture camera generated an additional textured image. Analysis of the resulting textured 3D images was performed using a software package (slim3D; 3D-Shape Ltd.) in accordance with a standardized workflow consisting of outlining the breast and calculating a back (chest) wall to complete the limits of the assumed breast volume, using a new nonlinear subdivision scheme. This calculation was carried out by two specially trained observers. Assessment of breast volumes has been previously reported in further detail by our group^19,20^.

### RANKL and OPG assessment

Serum samples in the CGATE study were frozen immediately after centrifugation and stored at –80 °C. Laboratory personnel were unaware of volume changes and had no access to the clinical data. Serum levels of soluble RANKL and OPG were measured using a sandwich and competitive enzyme immunoassay (Biomedica, Vienna, Austria), as previously described. According to the manufacturer, both the intra-assay and inter-assay coefficients of variation were lower than 10%, with detection limits of 0.14 pmol/L for soluble OPG and 0.08 pmol/L for soluble RANKL^21–23^.

### Compliance with ethical standards

This study was approved by the Ethics Committee of the Medical Faculty at Friedrich Alexander University of Erlangen–Nuremberg, Erlangen, Germany. All of the investigations complied with national law and with the 1975 Declaration of Helsinki in its current revised version. Written informed consent was obtained from all individual patients included in the study.

### Statistical analysis

The primary aim of this study was to explore whether RANKL or OPG influence breast volume at gestational week 40 in addition to established predictors. A linear regression model was therefore fitted with breast volume at gestational week 40 as the outcome and with the following predictors: age, body mass index (BMI) at gestational week 12, breast volume at gestational week 12, and gravida number. Next, another linear regression model was fitted with the same predictors as above, but additionally with RANKL (>0 versus = 0) and OPG (>0 versus = 0). The two regression models were compared using an F test. A significant test result means that the breast volume at week 40 was influenced by RANKL or OPG in addition to the other predictors considered.

Missing predictor values were imputed, as done by Salmen *et al*.^24^. Over-influential observations determined by Cook’s distance statistic were excluded. The coefficient of determination, *R*^2^, was calculated to measure the goodness of fit. The *R*^2^ coefficient was also internally validated using 10-fold cross-validation with 20 replications to address overfitting. The assumption of normally distributed standardized residuals was checked using the Shapiro–Wilk normality test and graphically using Q–Q plots, as previously described^20^.

A *P* value <0.05 was regarded as statistically significant. Calculations were carried out using the R system for statistical computing (version 3.4.0; R Development Core Team, Vienna, Austria, 2017).

## Results

**Figure 1:**
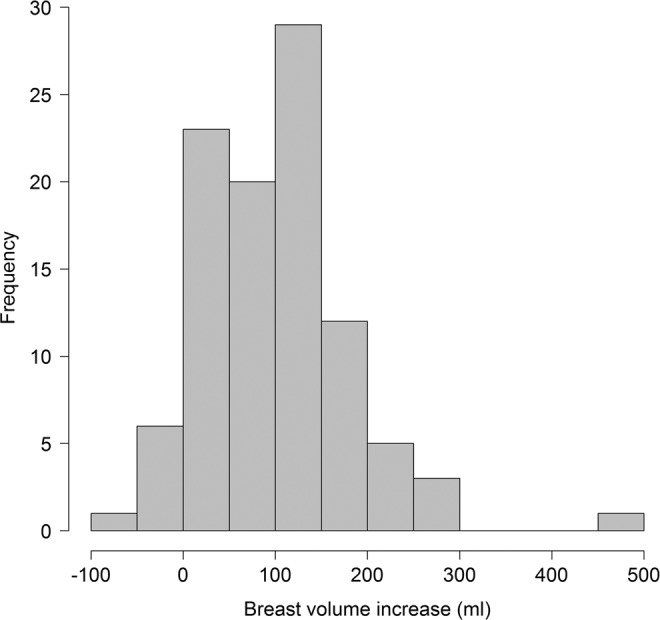
Distribution of changes in breast volume (mL) between gestational week 12 and 40 (week 40 minus week 12).

The mean age of the patients included in the study was 32. Most of the women were pregnant with either their first child (n = 35; 35%) or second child (n = 47, 47%). The mean breast volume at the start of pregnancy was 413.1 mL (standard deviation, SD 226.7 mL) and the mean volume at the end of pregnancy was 511.6 mL (SD 245.6 mL). The distribution of volume changes during pregnancy is shown in Fig. 1. Additional patient characteristics are listed in Table 1.

**Table 1:** Patient characteristics.

Characteristic	Mean or n	SD or %
Age (years)	32.2	4.2
BMI (kg/m^2^)	23.5	3.9
Breast volume at week 12 (mL)	413.1	226.7
Breast volume at week 40 (mL)	511.6	245.6
**Gravida**		
1	35	35.0
2	47	47.0
3	10	10.0
4	5	5.0
5	3	3.0
**RANKL**		
0	65	65.0
>0	35	35.0
**OPG**		
0	62	62.0
>0	38	38.0

Mean and standard deviation (SD) are shown for continuous characteristics, and frequency and percentage for categorical characteristics.

**Figure 2:**
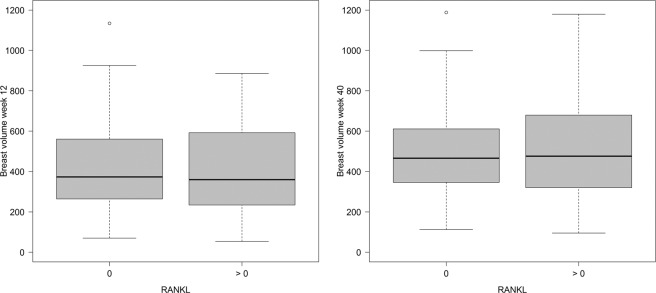
Breast volume (mL) (**a**) at gestational week 12 and (**b**) at gestational week 40, relative to RANKL status.

**Figure 3:**
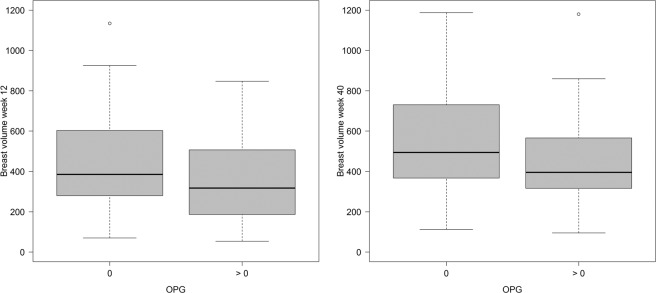
Breast volume (mL) (**a**) at gestational week 12 and (**b**) at gestational week 40, relative to OPG status.

At gestational week 12, RANKL was detectable in the serum of 35 women (35%) and OPG was detectable in the serum of 38 women (38%). In women with one pregnancy, 11 out of 35 subjects (31%) had measurable RANKL and 12 out of 35 subjects (34%) had measurable OPG in the serum. In women with more than one pregnancy, these numbers were 24 out of 65 (37%) and 26 out of 65 (40%). The distribution of the two markers relative to each other is shown in Table 2. RANKL serum values did not appear to have an influence on breast volumes at week 12 or week 40 (Fig. 2a,b), while women with detectable OPG in the serum appeared to have lower breast volumes than pregnant women who did not have detectable OPG at either point (Fig. 3a,b).

**Table 2:** Cross-table comparing detection of RANKL with detection of OPG.

	OPG = 0	OPG > 0
RANKL = 0	45	20
RANKL > 0	17	18

**Figure 4:**
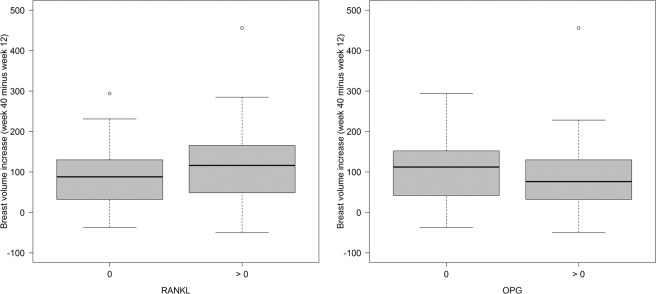
Increases in breast volume (mL) between gestational week 12 and 40, (**a**) relative to RANKL status and (**b**) relative to OPG status.

RANKL or OPG influenced breast volume at gestational week 40 in addition to the predictors considered (*P* = 0.04, F test). Regression coefficients are shown in Table 3. Breast volume at week 12 was the most important predictor, but BMI also had a negative influence on the increase in breast volume (Table 3). Patients with detectable RANKL values had greater increases in breast volume between week 12 and week 40 than those who did not have detectable RANKL (Table 3, Fig. 4a). Patients with detectable OPG had smaller volume increases than pregnant women who did not have measurable OPG (Table 3, Fig. 4b). The estimated mean breast volumes at week 40 relative to RANKL status and OPG status are shown in Table 4. Women who did not have measurable RANKL but did have detectable OPG had the lowest breast volumes, at 445 mL, while women with detectable RANKL and no OPG had an average breast volume of 504 mL.

**Table 3:** Linear regression model for predicting breast volume at gestational week 40.

Coefficient	Estimate	Standard error	*P* value
Intercept	205.03	66.80	—
Age, per year	0.77	1.78	0.66
BMI, per kg/m^2^	−6.70	2.25	<0.01
Gravida, per pregnancy	1.75	8.98	0.85
Breast volume at week 12, per mL	1.05	0.04	<0.000001
RANKL: > 0 versus 0	31.57	15.20	0.04
OPG: > 0 versus 0	−27.19	15.02	0.07

**Table 4:** Estimated breast volume (mL) at gestational week 40, with 95% confidence intervals, relative to RANKL and OPG status.

	OPG = 0	OPG > 0
RANKL = 0	472 (452, 492)	445 (419, 471)
RANKL > 0	504 (476, 532)	477 (449, 504)

A linear regression model was used to estimate breast volume at week 40 in an “average” patient, defined as a woman of median age (32 years), with a median body mass index (22.5 kg/m^2^), with the most frequent gravida number (2) and with a median breast volume at week 12 (371 mL).

**Figure 5:**
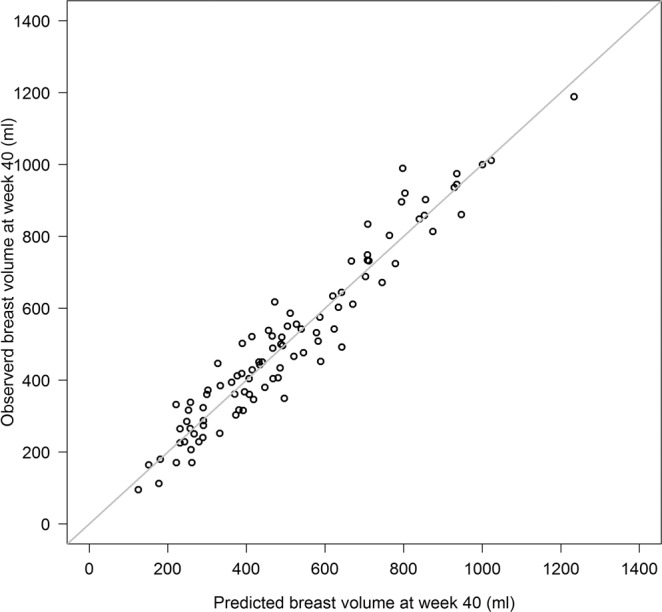
Predicted and observed breast volumes (mL) at gestational week 40 using the linear regression model.

The predicted and observed breast volumes at gestational week 40 are shown in Fig. 5. The coefficient of correlation *R*^2^ for the complete dataset was 0.93 and the cross-validated *R*^2^ was 0.88, indicating a small amount of overfitting.

## Discussion

This analysis of prospectively assessed breast volumes in healthy women at the start and the end of pregnancy shows that RANKL has a positive effect on breast volume increase and OPG has a negative influence. This may be the first study to link this important pathway to changes in the human breast during pregnancy.

Mammary development has been the focus of research projects for decades, and a variety of molecular pathways have been linked to neonatal, prepubertal, pubertal, pregnancy, and lactation-related changes^25–27^. However, most of the pathways described have been discovered and investigated in animal models — mainly mouse models — and there is a lack of confirmation studies in humans, due to the obvious restrictions on research involving underage individuals and pregnant women.

Trying to establish measurable phenotypes of breast changes during pregnancy, our group previously developed a surface-based 3D method of measuring the female breast^19^ and validated the method in a cohort of pregnant women^20^. Validation of a method of calculating mammographic density using ultrasound^28^ in a cohort of pregnant women is still ongoing. There have been a few earlier studies that had similar aims with the technical methods that were available in the 1990s^29,30^. In a small series of eight women who were prospectively assessed with regard to breast changes during pregnancy, plasma human placental lactogen was found to correlate with breast growth, but not plasma prolactin^29^. Since then, more than a hundred genes have been shown to regulate different aspects of mammary development, some of them overlapping with genes that have been identified as varying during the course of pregnancy^31^.

RANKL/RANK/OPG signalling is of particular importance in this context, as it has been identified — in addition to prolactin and parathyroid hormone-related peptide — as being one of the key regulators of mammary gland development during pregnancy^26,27,32,33^. In animal models, knockout of RANKL and RANK results in a lactational defect due to absent formation of a lactating mammary gland^33^. The importance of RANKL/RANK in this process also emphasizes the role of progesterone. Specifically, progesterone is reported to be the initiator of mammary epithelial proliferation through the induction of RANKL expression in progesterone receptor–positive breast epithelial cells. RANKL induces the proliferation of RANK-expressing mammary epithelial progenitor cells, which are mainly hormone receptor–negative^13,14,32,34^.

Our study now links RANKL and OPG measurements to changes in the breast during pregnancy, and hence supports further investigation of this phenotype in the context of its influence on breast cancer risk, which has not been studied so far. Assuming that RANKL supports proliferation of the breast during pregnancy, the observed effect in our study points in the right direction. OPG as a negative regulator of this pathway also showed an effect in the opposite direction. Thus, it may be hypothesized that the regulation of mammary development during pregnancy in healthy women is regulated in both directions by RANKL/RANK/OPG signalling.

It has been reported by our own group and others that pregnancies can have a substantial influence on breast density^5,12^. Our group also previously reported a link between serum calcium and mammographic density in patients with breast cancer^18^, and mammographic density has been connected with the RANKL/RANK/OPG pathway in a small study^35^. Evidence is therefore growing that breast phenotypes are associated with calcium metabolism and RANKL/RANK/OPG as the underlying molecular pathway.

This study suggests that RANKL/RANK/OPG signalling plays a role in breast changes during human pregnancies. It has already been shown that this pathway is specifically part of the pathogenesis of breast cancer in *BRCA1* mutation carriers^34,36,37^. In a small cohort study of *BRCA1/2* mutation carriers, high OPG serum levels have been shown to have a protective effect in relation to the development of breast cancer^38^. Treatment with denosumab has also been reported to reduce the proliferation of breast epithelial cells in surgical specimens from *BRCA1* mutation carriers^37^. A trial examining denosumab as a chemopreventive drug in *BRCA1* mutation carriers independently of pregnancy is currently ongoing^39^. Additional information about the molecular mechanisms involved in the way in which pregnancy influences breast tissue may therefore be useful for breast cancer prevention^1,34,40^. Identifying women who are at increased risk for breast cancer after pregnancy^4^ may help address this specific risk during that time period.

Participants of our study were healthy pregnant women, which were not selected specifically for a family history of breast cancer. Germline genetic testing for a *BRCA1/2* mutation was not performed. However, with regard to the low prevalence of *BRCA1/2* mutations, which is suspected to be 1/300–1/500 in the general population^41^, it is likely that all or nearly all participating women did not have a *BRCA1/2*-germline mutation. Breast volume changes and serum RANKL/RANK/OPG during pregnancy in *BRCA1/2*-mutation carriers vs. non-mutation carriers could be explored in future studies.

The present study has several strengths and limitations. It is a prospective study that aimed to identify molecular pathways that influence breast changes during pregnancy. 3D surface assessment^19^ is a noninvasive and easily performed method that could be used for future molecular studies of breast changes during pregnancy. One limitation of this study is its small sample size. However, the effects of RANKL and OPG in the expected directions may allow some confidence in the results. In addition, no association between breast cancer risk and breast volume changes during pregnancy has so far been reported.

The clinical relevance of the present results thus remains unclear. Nonetheless, women with a high RANKL and a low OPG serum level at the beginning of the pregnancy seem to have an average of 59 mL larger breast at the end of the pregnancy than women with a low RANKL and a high OPG serum level. It could be hypothesized that pregnancy in these two groups of women has a different effect on the breast after pregnancy, which has to be further investigated. Moreover, to examine changes of the breast with regard to breast cancer risk and other phenotypes, such as predicted breast density by ultrasound^28^, might be helpful.

In conclusion, this study provides the first confirmation that RANKL and OPG play a role in breast changes in healthy women during pregnancy, with serum RANKL being linked to a greater increase in breast volume and OPG being associated with a smaller increase on breast volume. Future studies will need to address the way in which these findings are linked to the risk of breast cancer and how this can be used for breast cancer prevention.

## Data Availability

The datasets generated and/or analysed during the current study are available from the corresponding author on reasonable request.

## References

[1] Fasching PA (2011). Breast Cancer Risk - Genes, Environment and Clinics. Geburtsh Frauenheilk.

[2] Wunderle M (2018). Risk, Prediction and Prevention of Hereditary Breast Cancer - Large-Scale Genomic Studies in Times of Big and Smart Data. Geburtshilfe Frauenheilkd..

[3] Collaborative Group on Hormonal Factors in Breast Cancer (2002). Breast cancer and breastfeeding: collaborative reanalysis of individual data from 47 epidemiological studies in 30 countries, including 50302 women with breast cancer and 96973 women without the disease. Lancet.

[4] Lambe M (1994). Transient Increase in the Risk of Breast Cancer after Giving Birth. N. Engl. J. Med..

[5] Hack CC (2017). Association between mammographic density and pregnancies relative to age and BMI: a breast cancer case-only analysis. Breast cancer Res. Treat..

[6] Heusinger K (2011). Mammographic density as a risk factor for breast cancer in a German case-control study. Eur. J. Cancer Prev..

[7] Vachon CM, Kuni CC, Anderson K, Anderson VE, Sellers TA (2000). Association of mammographically defined percent breast density with epidemiologic risk factors for breast cancer (United States). Cancer Causes Control..

[8] Gram IT, Funkhouser E, Tabar L (1995). Reproductive and menstrual factors in relation to mammographic parenchymal patterns among perimenopausal women. Br. J. Cancer.

[9] Bartow SA, Pathak DR, Mettler FA, Key CR, Pike MC (1995). Breast mammographic pattern: a concatenation of confounding and breast cancer risk factors. Am. J. Epidemiol..

[10] Kaufman Z, Garstin WI, Hayes R, Michell MJ, Baum M (1991). The mammographic parenchymal patterns of nulliparous women and women with a family history of breast cancer. Clin. Radiol..

[11] Rauh C (2012). Percent Mammographic Density and Dense Area as Risk Factors for Breast Cancer. Geburtsh Frauenheilk.

[12] Loehberg CR (2010). Assessment of mammographic density before and after first full-term pregnancy. Eur. J. Cancer Prev..

[13] Asselin-Labat ML (2010). Control of mammary stem cell function by steroid hormone signalling. Nature.

[14] Joshi PA (2010). Progesterone induces adult mammary stem cell expansion. Nature.

[15] Schramek D (2010). Osteoclast differentiation factor RANKL controls development of progestin-driven mammary cancer. Nature.

[16] Gonzalez-Suarez E (2010). RANK ligand mediates progestin-induced mammary epithelial proliferation and carcinogenesis. Nature.

[17] Heusinger K (2012). Association of mammographic density with hormone receptors in invasive breast cancers: results from a case-only study. Int. J. Cancer.

[18] Hack CC (2017). Correlation of mammographic density and serum calcium levels in patients with primary breast cancer. Cancer Med..

[19] Koch MC (2011). Breast volumetry using a three-dimensional surface assessment technique. Aesthetic plastic Surg..

[20] Bayer CM (2014). Assessment of breast volume changes during human pregnancy using a three-dimensional surface assessment technique in the prospective CGATE study. Eur. J. Cancer Prev..

[21] Kiechl S (2017). Aberrant regulation of RANKL/OPG in women at high risk of developing breast cancer. Oncotarget.

[22] Kiechl S (2004). Osteoprotegerin is a risk factor for progressive atherosclerosis and cardiovascular disease. Circulation.

[23] Kiechl S (2007). Soluble receptor activator of nuclear factor-kappa B ligand and risk for cardiovascular disease. Circulation.

[24] Salmen J (2014). Pooled analysis of the prognostic relevance of progesterone receptor status in five German cohort studies. Breast cancer Res. Treat..

[25] Hassiotou F, Geddes D (2013). Anatomy of the human mammary gland: Current status of knowledge. Clin. Anat..

[26] Hennighausen L, Robinson GW (2001). Signaling pathways in mammary gland development. Dev. Cell.

[27] Hennighausen L, Robinson GW (2005). Information networks in the mammary gland. Nat. Rev. Mol. Cell Biol..

[28] Jud SM (2012). Correlates of mammographic density in B-mode ultrasound and real time elastography. Eur. J. Cancer Prev..

[29] Cox DB, Kent JC, Casey TM, Owens RA, Hartmann PE (1999). Breast growth and the urinary excretion of lactose during human pregnancy and early lactation: endocrine relationships. Exp. Physiol..

[30] Daly SE (1992). The determination of short-term breast volume changes and the rate of synthesis of human milk using computerized breast measurement. Exp. Physiol..

[31] Aghaeepour N (2018). A proteomic clock of human pregnancy. Am. J. Obstet. Gynecol..

[32] Tanos T (2013). Progesterone/RANKL is a major regulatory axis in the human breast. Sci. Transl. Med..

[33] Fata JE (2000). The osteoclast differentiation factor osteoprotegerin-ligand is essential for mammary gland development. Cell.

[34] Sigl Verena, Jones Laundette P., Penninger Josef M. (2016). RANKL/RANK: from bone loss to the prevention of breast cancer. Open Biology.

[35] Moran O (2018). Serum osteoprotegerin levels and mammographic density among high-risk women. Cancer Causes Control..

[36] Sigl V (2016). RANKL/RANK control Brca1 mutation-driven mammary tumors. Cell Res..

[37] Nolan E (2016). RANK ligand as a potential target for breast cancer prevention in BRCA1-mutation carriers. Nat. Med..

[38] Oden L (2016). Plasma osteoprotegerin and breast cancer risk in BRCA1 and BRCA2 mutation carriers. Oncotarget.

[39] Francis, P., Singer, C., Saunders, C., Garber, J. & Lindeman, G. J. BRCA-P: An international randomised phase III study evaluating the RANK ligand inhibitor denosumab for the prevention of breast cancer in BRCA1 mutation carriers [2018–2022], http://purl.org/au-research/grants/nhmrc/1140715 and, https://researchdata.ands.org.au/brca-p-an-mutation-carriers/1319302 accessed April 28, 2019.

[40] Schneeweiss A (2018). Update Breast Cancer 2018 (Part 2) - Advanced Breast Cancer, Quality of Life and Prevention. Geburtshilfe Frauenheilkd..

[41] King MC, Levy-Lahad E, Lahad A (2014). Population-based screening for BRCA1 and BRCA2: 2014 Lasker Award. JAMA.

